# Oxytocin Increases the Perceived Value of Both Self- and Other-Owned Items and Alters Medial Prefrontal Cortex Activity in an Endowment Task

**DOI:** 10.3389/fnhum.2017.00272

**Published:** 2017-05-23

**Authors:** Weihua Zhao, Yayuan Geng, Lizhu Luo, Zhiying Zhao, Xiaole Ma, Lei Xu, Shuxia Yao, Keith M. Kendrick

**Affiliations:** Key Laboratory for Neuroinformation, Center for Information in Medicine, School of Life Science and Technology, University of Electronic Science and Technology of ChinaChengdu, China

**Keywords:** endowment effect, self-processing, oxytocin, medial prefrontal cortex, value

## Abstract

The neuropeptide oxytocin (OXT) can influence self-processing and may help motivate us to value the attributes of others in a more self-like manner by reducing medial prefrontal cortex (mPFC) responses. We do not know however whether this OXT effect extends to possessions. We tend to place a higher monetary value on specific objects that belong to us compared to others, known as the “endowment effect”. In two double-blind, between-subject placebo (PLC) controlled experiments in subjects from a collectivist culture, we investigated the influence of intranasal OXT on the endowment effect, with the second study incorporating functional magnetic resonance imaging (fMRI). In the task, subjects decided whether to buy or sell their own or others’ (mother/father/classmate/stranger) possessions at various prices. Both experiments demonstrated an endowment effect in the self-owned condition which extended to close others (mother/father) and OXT increased this for self and all other-owned items. This OXT effect was associated with reduced activity in the ventral mPFC (vmPFC) in the self-owned condition but increased in the mother-condition. For the classmate- and stranger-owned conditions OXT increased activity in the dorsal mPFC (dmPFC). Changes in vmPFC activation were associated with the size of the endowment effect for self- and mother-owned items. Functional connectivity between the dmPFC and ventral striatum (VStr) was reduced by OXT in self- and mother-owned conditions and between vmPFC and precuneus in the self-condition. Overall our results show that OXT enhances the endowment effect for both self- and other-owned items in Chinese subjects. This effect is associated with reduced mPFC activation in the self-condition but enhanced activation in all other-conditions and involves differential actions on both dorsal and ventral regions as well as functional connectivity with brain reward and other self-processing regions. Overall our findings suggest that OXT increases the perceived value of both self- and other-owned items by acting on neural circuitry involved in self-processing and reward.

## Introduction

The hypothalamic neuropeptide oxytocin (OXT) has increasingly been shown to influence aspects of social cognition, affiliative behavior and social bonds (Bartz et al., 2011; Bethlehem et al., 2013). Evidence also suggests that OXT affects high level cognitive appraisal processes, resulting in the more positive and less negative appraisals of socially-relevant information concerning both self and others (Guastella et al., 2009; Guastella and MacLeod, 2012). Some initial studies suggested that intranasal OXT administration may increase self-perception due to promoting positive self-referential processing (Cardoso et al., 2012) and the ability to discriminate between their own face (self) and that of an unfamiliar person (other; Colonnello et al., 2013). However, more recent studies have found that it increases taking another, but not a self-perspective, in the context of pain perception (Abu-Akel et al., 2015) and other- but not self-orientation in terms of subjects perceiving themselves as being more communal (Bartz et al., 2015). We have also shown that OXT appears to blur the distinction between self and others in terms of trait-judgments and reduced medial prefrontal cortex (mPFC) responses as well as reduced functional connectivity with other regions of the default mode network (DMN) involved in self-processing (Zhao et al., 2016). These latter studies indicating that OXT may actually reduce self-interest resonate more closely with its well established role in promoting affiliative behavior and social bonds (Bartz et al., 2011; Bethlehem et al., 2013). However, it is unclear whether this OXT-effect in reducing self-interest extends beyond the immediate social domain of enhanced responses to the social importance of others, and the emotional behaviors and attributes they exhibit, to a more non-social one such as possessions.

We always place greater value on items that we actually own and this is referred to as the endowment effect (Thaler, 1980; Kahneman et al., 1990, 1991). In other words, we assign higher value and desirability ratings for self-owned objects compared with same ones which are owned by others. The endowment effect is measured in terms of the difference between willingness to accept (WTA) a price for an object and willingness to pay for it (WTP), and WTA is always higher than WTP (Procaccia and Segal, 2003). Loss aversion has traditionally been used to explain the endowment effect which makes buyers frame goods as gains but sellers as losses, and people consider loss as greater than an equivalent gain (Morewedge et al., 2009; Morewedge and Giblin, 2015). However, a number of studies have reported that the endowment effect can also be viewed as an exemplar of a mere ownership effect whereby a target object is rated more favorably by an owner than a non-owner (Beggan, 1992; Gawronski et al., 2007) and a recent review of the field has also emphasized this aspect (Morewedge and Giblin, 2015). One’s sense of self can therefore extend beyond the sense of body ownership and agency (Aron et al., 1991, 2004), to include one’s possessions and these can therefore be considered as part of one’s extended self (Belk, 1988). Because of the intrinsic tendency to enhance one’s self, the association between possession and self (ownership) subsequently boosts the possession’s perceived value (Ebisch et al., 2011; Dommer and Swaminathan, 2013). Our tendency to emphasize our positive self-characteristics is also relevant in this respect and there is evidence that OXT can enhance this positivity (Cardoso et al., 2012). This concept of one’s sense of self being extended to include possessions is further supported in the context of the endowment effect by our recent study showing that in members of a collectivist culture (Chinese) where the sense of self is extended to include close others (mother), this also extends to placing greater value on their possessions (Feng et al., 2013). Based on previous experiments indicating that OXT reduces self-interest by increasing consideration of others we therefore hypothesized that in Chinese subjects it would either selectively reduce the endowment effect for self- and mother-owned objects or alternatively extend it to include objects owned by others not normally included in the extended self (i.e., colleagues or strangers).

The mPFC has been implicated in seemingly disparate cognitive functions, such as understanding the minds of other people, processing information about the self, mental states, physical characteristics (Mitchell et al., 2005; Cikara et al., 2014), self-reflection, person perception and making inferences about others’ thoughts (Amodio and Frith, 2006). In an imagined ownership paradigm, the mPFC showed greater activity for self-owned objects compared with other-owned ones. Additionally, mPFC activation is increased for self- vs. other-owned objects and recognition memory for self-owned objects is correlated with this (Kim and Johnson, 2010, 2013). Increased mPFC activation has also been found in association with the endowment effect (Knutson et al., 2008; Feng et al., 2013). Taken together, these findings provide evidence that the mPFC is involved in the incorporation of self-relevant objects into one’s sense of self. Thus we hypothesized that since OXT decreases the activity of both dorsal and ventral regions of the mPFC during self-processing involving trait-judgments (Zhao et al., 2016), it would have a similar impact in relation to the self-bias associated with possessions.

We have therefore investigated the effects of intranasal OXT effect on self-processing in the context of the endowment effect in two independent studies, the second of which was combined with functional magnetic resonance imaging (fMRI) to investigate the neural substrates involved. Performing two independent studies also allowed us establish the general reproducibility of any observed behavioral effects of OXT and additionally whether two different doses of OXT which have previously been shown to produce functional effects (24 and 40IU—see Striepens et al., 2011; Guastella et al., 2013) might have a different efficacy. In our previous study using Chinese subjects (Zhao et al., 2016) we found evidence using a trait-judgment paradigm both for an extended sense of self incorporating close relatives such as mother, and that OXT effects were moderated by levels of self-esteem. In the current study, we therefore also included ownership of objects by both close (mother/father) and more remote (classmate/stranger) others and investigated potential moderating effects of self-esteem.

## Materials and Methods

Two independent, double-blind, between subjects, placebo (PLC) controlled design studies were performed on different groups of subjects. The first study (Experiment 1) involved a behavioral analysis of the influence of OXT on the endowment effect using a lower functional dose (24IU) while in a second study (Experiment 2) OXT effects on the endowment effect were investigated during simultaneous fMRI scanning and using a higher functional dose (40IU).

### Experiment 1

#### Participants

Subjects randomly assigned to OXT and PLC treatment groups and tested individually. A total of 35 male Chinese subjects (mean age ± SEM = 21.29 ± 0.31 years) were included. The subjects were all University students and were free of medical or psychiatric illness, drug or alcohol abuse. It was confirmed that all subjects had both parents living and had a good relationship with them. The study was approved by the ethical committee of the University of Electronic Science and Technology of China and all subjects gave written informed consent to take part in accordance with the latest revision of the Declaration of Helsinki. Subjects’ privacy rights were always observed.

For the experiment, subjects were first administered a single intranasal dose of the lower OXT dose (24 IU, OXT Spray—Sichuan Meike Pharmacy Co. Ltd., Sichuan, China; three puffs of 4IU per nostril with 30 s between each puff) or PLC (also three puffs per nostril) using a standard protocol (Guastella et al., 2013). The PLC treatment was also provided in the same type of dispenser bottle by the pharmaceutical supply company providing the OXT nasal spray, and contained all of same ingredients (sterile water, sodium chloride and glycerol) other than the neuropeptide. In line with many previous reports and the standard recommended protocol experimental paradigm (Guastella et al., 2013) started 45 min after OXT or PLC treatment and lasted for around another 40 min. It should be acknowledged that studies have reported variable time-courses for increased cerebrospinal fluid concentrations of OXT after intranasal application in either monkeys or humans (Chang et al., 2012; Guastella et al., 2013). However, a recent study measuring regional cerebral blood flow changes in the human brain following intranasal OXT also reported extensive changes in brain regions with OXT receptors at 39–51 min after treatment (Paloyelis et al., 2016). In post-experiment interviews, subjects were unable to identify better than chance whether they had received the OXT or PLC treatment.

Immediately before the experiment all subjects completed a range of questionnaires measuring personality and affective traits and levels of anxiety: Chinese versions of: NEO-Five Factor Inventory (NEO-FFI; Costa and Mccrae, 1989), Positive and Negative Affect Schedule (PANAS; Watson et al., 1988), State-Trait Anxiety Inventory (STAI; Spielberger et al., 1970), Self-Esteem Scale (SES; Rosenberg, 1989) and Inclusion of Others in Self (IOS; Aron et al., 1992).

#### Experimental Design

Subjects participated in a modified version of the Savings Hold Or Purchase (SHOP) task (Knutson et al., 2008; Feng et al., 2013). In this task subjects are repeatedly presented with a small number of different items and informed on each occasion whether they are owned by themselves or others. The identical objects are displayed in each of the different ownership conditions so that the only variable is its ownership. For each presentation they are then given different suggested prices to either buy or sell the object and in each case they have to decide whether to accept or not. For each item and its ownership an indifference point is calculated for the price at which the subject is prepared to sell (what price to accept—WTA) or buy (what price to purchase—WTP). The size endowment effect for self is calculated as the indifference point for WTA minus that for WTP. The endowment effect for other ownerships is calculated relative to that for self and is thus calculated as the WTA indifference point for the owner minus the WTP for self in each case.

In the current study prior to the task, subjects were shown three different common personal item products but without being informed about their specific retail purchase prices (watch—high price; vacuum flask—medium price and pen—low price). Subjects were then also asked to rate their preference for the three items on a 1–7 Likert scale. Note that in Chinese culture small vacuum flasks are a personal possession used every day for drinking hot water or tea. The two tasks were programmed and presented using E-Prime version 2.0 (Psychology Software Tools, Inc., Sharpsburg, PA, USA).

**Figure 1:**
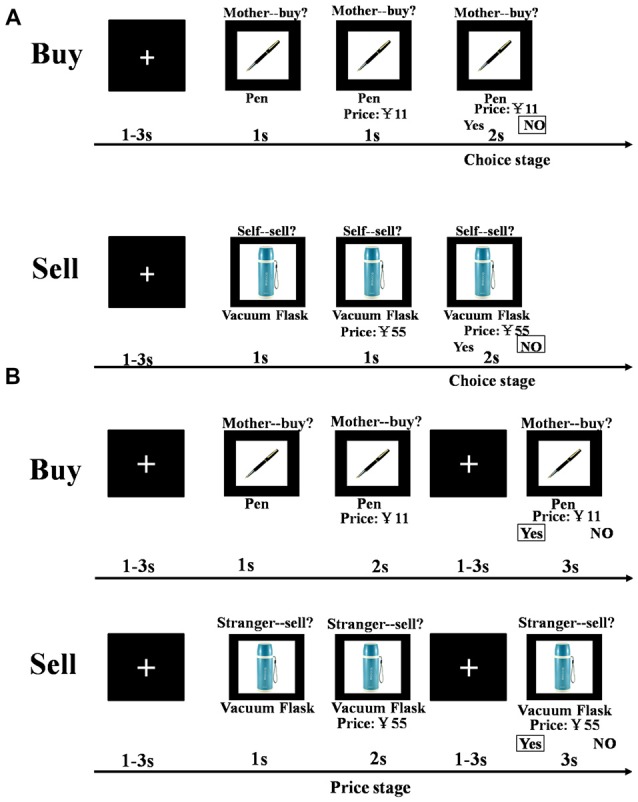
**Schematic representation of the task paradigm (Behavior and fMRI). (A)** Following a 1–3 s jittered white fixation on a black background, a target item was presented for 1 s and another 1 s with price labeled below. Then subjects were asked to make a decision in 2 s about whether to buy (sell) at the displayed price or not. **(B)** Following a 1–3 s jittered white fixation on a black background, a target item was presented for 1 s and another 2 s with price labeled below. Then subjects were asked to make a decision in 2 s about whether to buy (sell) at the displayed price or not followed by another jittered 1–3 s fixation.

The Buy and Sell sessions were structured identically. Each trial consisted of a presentation of a fixation cross ranging from 1 s to 3 s (jittered), followed by a target item presented for 1 s and then another presentation of the target together with a price for 1 s. The prices used in the stimuli were evenly distributed from 5% to 95% of the value of an item (12 prices: 5%, 15%, 25%, 35%, 40%, 45%, 55%, 60%, 65%, 75%, 85%, 95%; where 50% was the actual retail price of the item). Participants were then given 2 s to decide whether or not they would buy or sell the product at the indicated price (for themselves, for their mother, for their father, or for their classmate; see Figure 1A).

In a pilot experiment on 14 male subjects (mean ± SEM age = 21.21 ± 2.01 years), we confirmed that the first likeability ratings for the three different personal items did not differ significantly analysis of variance (ANOVA—*F*_(2,26)_ = 0.90, *p* = 0.42) and second that the size of the endowment effect in self and other ownership conditions did not differ significantly across the three objects (ANOVA—*F*_(2,26)_ = 0.08, *p* = 0.92). For analysis we therefore combined data from the three different items to increase statistical power.

### Experiment 2

#### Participants

In the same overall design as in Experiment 1, subjects were randomly assigned to OXT and PLC treatment groups. A total of 41 male subjects (mean age ± SEM = 22.83 ± 0.34 years) participated in a combined behavior and fMRI experiment (two subjects were excluded due to excessive head movement leaving 20 in the OXT group and 19 in the PLC group). It was confirmed that all subjects had both parents living and had a good relationship with them. All subjects were right handed. The study was also approved by the ethical committee of the University of Electronic Science and Technology of China and all subjects gave written informed consent to take part in accordance with the latest revision of the Declaration of Helsinki. Subjects’ privacy rights were always observed.

Subjects in the OXT group were administered the higher routine OXT dose of 40IU (five puffs of 4IU per nostril with 30 s between each puff) or PLC (also five puffs per nostril). The experimental paradigm started 45 min after OXT or PLC treatment as in Experiment 1. In post-experiment interviews subjects were unable to identify better than chance whether they had received the OXT or PLC treatment. Before treatment subjects also completed NEO-FFI, PANAS, STAI, SES and IOS questionnaires and additionally the Self-Construal Scale (SCS; Singelis, 1994).

#### Experimental Design

The procedure was similar to Experiment 1 except for small timing modifications to aid with the fMRI analysis. Thus, the duration of presentation of the target item with a price was increased from 1 s to 2 s and an additional presentation of the fixation cross was then included for a jittered 1–3 s, before participant were given 3 s to decide whether or not they would buy or sell the product at the indicated price (for themselves, their mother, their classmate or a stranger; see Figure 1B). As in Experiment 1 all items were rated by subjects for likeability prior to treatment. During the task all subjects responded in the MRI scanner using two fMRI compatible 4-key button boxes (one for each hand) by pressing the first key (left hand side of each box) using the index finger on either their left or right hand to indicate “yes” or “no”. The position of the “yes” and “no” on the left and right hand side underneath the object and its price indicated which hand to use to make a response, and was random. Thus, in half the trials a “yes” response involved a button press by the index finger of the left hand and in the other half of the trials it involved the index finger of the right hand, and vice versa for a “no” response. As in Experiment 1 tasks were programmed and presented using E-Prime version 2.0 (Psychology Software Tools, Inc., Sharpsburg, PA, USA).

Stimuli were presented in four runs of 36 trials each (two runs for the sell condition and two runs for the buy condition) and the order of the conditions (buy/sell) was balanced. In the sell condition, 36 trials (12 prices * 3 items) were included for each type of ownership. Thus in total, there were 144 trials (i.e., 36 * 4) in each condition and the trials were randomized. As in Experiment 1 for each subject the overall size of the endowment effect and likeability ratings were calculated across the three items combined.

### Behavioral Data Analysis

Statistical analyses of data were performed with SPSS® 22.0 statistical software package (SPSS Inc., Chicago, IL, USA). Initially, paired *t*-tests were used to establish whether there was a significant endowment effect in each of the four ownership conditions within the two treatment groups. Next, two-way repeated ANOVAs with treatment and ownership as factors were used to investigate significant differences between treatment groups and different ownership conditions. Pearson correlation was used to test for significant correlations between endowment effect sizes and trait scores and neural activity and functional connectivity parameters with Fisher’s *z* being used to test for significance between correlations in the two treatment groups. Effect sizes were also calculated using either partial-eta squared or Cohen’s *d* as appropriate. All statistical tests were two-tailed and *p* < 0.05 was considered statistically significant. In all cases a Bonferroni correction was made for multiple comparisons.

### fMRI Acquisition

A high-resolution T1-weighted structural volume was acquired using a GE (General Electric Medical System, Milwaukee, WI, USA) 3.0T MRI scanner to measure changes in blood oxygenation level-dependent (BOLD) activity. During each fMRI scan, a time series of volumes was acquired using a T2*-weighted echo planar imaging pulse sequence (repetition time, 200 ms; echo time 30 ms; slices, 39; thickness, 4 mm; gap, 1 mm; field of view, 240 × 240 mm^2^; resolution, 64 × 64; flip angle, 90°). The 3D spoiled gradient recalled (SPGR) sequence used the following parameters: repetition time, 6 ms; echo time, 2 ms; flip angle, 9°; field of view, 256 × 256 mm^2^; acquisition matrix, 256 × 256; thickness, 1 mm without gap.

### fMRI Data Processing

fMRI data were analyzed using SPM8 (Wellcome Department of Cognitive Neurology, London, UK^1^; Friston et al., 1994). Slice timing was used to correct slice order, images were realigned to correct for head movement based on a six-parameter rigid body algorithm and the first five images were discarded to achieve magnet-steady images. These images were then normalized to MNI space in 3 mm × 3 mm × 3 mm voxel sizes. The normalized data were spatially smoothed with a Gaussian kernel; the full width at half maximum (FWHM) was specified as 8 mm × 8 mm × 8 mm. After pre-processing, 10 regressors (buy for self, buy for mother, buy for classmate, buy for stranger, sell for self, sell for mother, sell for classmate, sell for stranger, product and choice) were modeled to create the design matrix. They were convolved with the canonical hemodynamic response function, and the six realignment parameters for each subject were also included as confounding factors. For all of the task-related analyses neural activity and functional connectivity changes were divided into two phases. The first (price stage) is defined as the difference between the initial fixation period (1–3 s jittered) for each trial and the presentation period of the item by itself and the item together with a suggested price (at total of 3 s). The second phase (response phase) is the difference between the second 1–3 s fixation and the 3 s period where subjects respond using the key press whether to accept the price for the item or not.

### Whole-Brain Analysis

The endowment effect results in sellers demanding more than buyers (Kahneman et al., 1990, 1991), thus in the current study we just focused on selling behavior in line with other previous studies (Feng et al., 2013; Tong et al., 2016). In the whole brain analysis, we identified brain regions whose activation was associated with OXT modulation of the endowment effect. In the task, we investigated main effects of ownership (self, mother, classmate, stranger) and interactions between ownership and treatment using ANOVA and explored main effects of treatment (OXT vs. PLC) using two-sample *t*-tests for all types of ownerships. The threshold for fMRI data significance was set to *p* < 0.05, false discovery rate (FDR) corrected, with a minimum cluster size of 10 contiguous voxels. The differences between two groups in the contrasts of self, mother, classmate, stranger; self vs. mother, self vs. classmate, self vs. stranger, self vs. remote others (classmate and stranger) were analyzed using two-sample *t*-tests. Since mPFC (dmPFC and vmPFC) activations are related to self-processing, they were defined as regions of interest (ROIs). A small volume correction (SVC) was also applied with the mPFC region as defined in the Wake Forest University (WFU) PickAtlas (Maldjian et al., 2003), and we thresholded the result using *p* < 0.05.

### Region of Interest (ROI) Analysis

Individual dorsal mPFC (dmPFC: −6, 46, 20) activation was extracted from an 8 mm radius sphere centered at co-ordinates previously identified in an independent sample (Wang et al., 2012). The co-ordinate for the 8 mm ventral mPFC sphere (vmPFC: −18, 56, 7) was chosen on the basis of our whole brain analysis in the self-ownership condition across the two groups. An additional 8 mm dmPFC sphere using co-ordinate (−15, 14, 52) was chosen on the basis of our whole brain analysis of self vs. remote others (classmate and stranger) across the two groups. These ROIs were all defined using MarsBar to extract neural activation for further analysis (correlated with behavioral performance) during the price stage (Brett et al., 2002). Pearson correlation analysis was used to determine associations between regional activation and behavior.

### Generalized Psychophysiological Interaction (gPPI) Analysis

Functional connectivity analysis was performed to investigate altered connectivity between different brain regions in the different ownership conditions (self/mother/classmate/stranger). We measured functional connectivity using a generalized form of context-dependent psychophysiological interactions (gPPI) analysis (Friston et al., 1994; McLaren et al., 2012; O’Reilly et al., 2012). Based on ROI results, we chose the two main mPFC ROIs (vmPFC and dmPFC) as seed regions to investigate functional connectivity. For the gPPI analysis, we extracted the de-convolved time-course of each seed region in each subject, based on an 8 mm radius sphere centered on the peak-activation voxel from the mPFC ROIs [dmPFC (−6, 46, 20), vmPFC (−18, 56, 7)]. We calculated the product of this activation time-course and the vector of the psychological variable of interest to create the psychophysiological interaction term. New SPMs were computed for each subject, including the interaction term, the physiological variable (i.e., the ROI activation time-course) and the psychological variable as regressors. We then identified areas where activation was predicted by the psychophysiological interaction term, with ROI activity and the psychological regressor treated as confound variables. These analyses were carried out separately for either self, mother, classmate or stranger ownership. Individual PPI SPMs were entered into a random-effects group analysis contrasting connectivity patterns in each ownership with two-sample *t*-tests (OXT vs. PLC), thresholded at *p* < 0.05, small volume corrected, with a minimum cluster size of 10 voxels. The strengths of functional connections between seed regions and the target region were also correlated with behavioral responses using a Pearson correlation analysis.

## Results

### Experiment 1

Table 1 shows that there were no significant differences between the OXT and PLC groups in terms of age or questionnaire scores. While there was no difference between likeability ratings for the three different items in the OXT and PLC groups (vacuum flask: *t*_(33)_ = 1.44, *p* = 0.16; watch: *t*_(33)_ = 1.15, *p* = 0.28; pen: *t*_(33)_ = 0.07, *p* = 0.95) in contrast to our pilot group finding subjects in both groups did show a significant difference (ANOVA—*F*_(2,68)_ = 6.09, *p* = 0.004) due to the pen being given lower likeability ratings than the vacuum-flask (*p* = 0.006) or watch (*p* = 0.018). There were no significant differences between IOS scores for mother, father and classmate in the two treatment groups. The high IOS scores in both groups for mother and father compared with those for classmate confirm previous findings that individuals in collectivist cultures have an extended self-concept which includes close family members (Zhu et al., 2007).

**Table 1:** **Age and questionnaire scores for 1^st^ Experiment (mean ± SEM)**.

Measurements	Placebo	Oxytocin	*t*-value	*p*-value
Age (years)	21.00 ± 0.40	21.53 ± 0.45	0.86	0.40
NEO-Five Factor Inventory (NEO-FFI)				
Neuroticism	33.25 ± 2.21	32.74 ± 1.86	0.18	0.86
Extraversion	41.88 ± 2.01	43.58 ± 1.19	0.76	0.46
Openness to experience	40.56 ± 1.01	39.68 ± 1.24	0.54	0.60
Agreeableness	42.19 ± 1.30	41.42 ± 0.84	0.51	0.61
Conscientiousness	43.38 ± 1.31	42.31 ± 0.94	0.67	0.51
Positive and Negative Affective Scale (Positive)	30.13 ± 1.89	30.31 ± 1.97	0.07	0.95
Positive and Negative Affective Scale (Negative)	17.75 ± 1.42	17.95 ± 1.45	0.10	0.92
State-Trait Anxiety Inventory (STAI)—State	39.75 ± 2.61	39.89 ± 2.47	0.04	0.97
State-Trait Anxiety Inventory (STAI)—Trait	45.50 ± 2.31	44.89 ± 2.00	0.20	0.84
Self-Esteem Scale (SES)	32.00 ± 1.00	31.32 ± 1.16	0.44	0.67
Inclusion of Others in Self (IOS)—Mother	6.31 ± 0.15	6.53 ± 0.19	0.85	0.40
Inclusion of Others in Self (IOS)—Father	5.50 ± 0.37	5.74 ± 0.25	0.55	0.59
Inclusion of Others in Self (IOS)—Classmate	3.00 ± 0.18	2.68 ± 0.23	1.05	0.30

As in previous studies, subjects showed a robust endowment effect in terms of the difference between WTA and WTP (Knutson et al., 2008; Feng et al., 2013). The mean indifference point in the sell condition (WTA) significantly exceeded that in the buy condition (WTP) for self- (*t*_(34)_ = 4.30, *p* < 0.001, Cohen’s *d* = 0.82), mother- [*t*_(34)_ = 5.51, *p* < 0.001, Cohen’s *d* = 1.17] and father- [*t*_(34)_ = 4.72, *p* < 0.001, Cohen’s *d* = 1.10] but not for classmate-owned [*t*_(34)_ = 0.15, *p* = 0.88] products across both groups. We confirmed our pilot study findings that there was no significant difference between the three items in terms of their contribution to the overall size of the endowment effect (ANOVA—*F*_(2,68)_ = 1.75, *p* = 0.19). These results show that the endowment effect extends from self to close others (father/mother), but not to more remote ones (classmate) in subjects from a collectivist culture.

**Figure 2:**
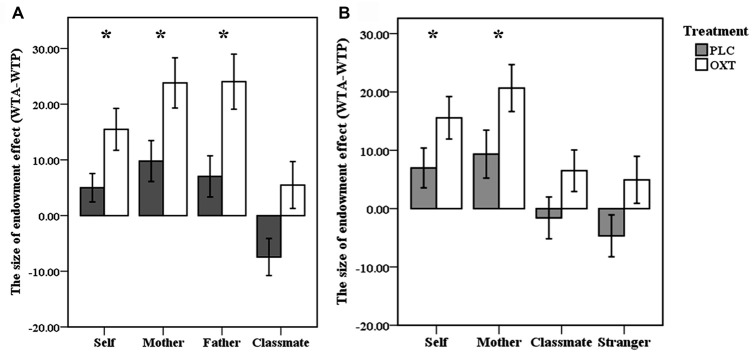
**Behavioral results. (A,B)** Mean indifference points between willingness to accept (WTA) and willingness to pay (WTP; percent retail price ± SEM), plotted as a function of the size of endowment effect for self, mother, father, classmate in oxytocin (OXT) and placebo (PLC) groups from the first experiment **(A)** and for self, mother, classmate and stranger in the second fMRI experiment **(B)**. *p < 0.05 for OXT vs. PLC.

To investigate whether OXT treatment modulated the endowment effect, a two-way ANOVA revealed a significant main effect of ownership [*F*_(3,99)_ = 25.63, *p* < 0.001, partial *η*^2^ = 0.437] and treatment [*F*_(1,33)_ = 7.56, *p* = 0.01, partial *η*^2^ = 0.19] but there was no interaction between them [*F*_(1,37)_ = 0.713, *p* = 0.546] (see Figure 2A). Thus, OXT enhanced the size of the endowment effect (WTA-WTP) across all types of ownerships.

There were no significant correlations between the size of the endowment effect in the two groups and self-esteem scores.

Based on the results of this initial behavioral study, we modified the endowment effect task to investigate the neural mechanism of OXT’s effect using fMRI by replacing the father ownership condition with a stranger ownership one in order to produce the same number of conditions with and without an endowment effect (i.e., 2 in each case).

### Experiment 2

#### Behavioral Results

Table 2 shows that there were no significant differences between the OXT and PLC groups in age or questionnaire scores. Once again there was no significant difference in likeability ratings for the three items between the two treatment groups (vacuum flask: *t*_(39)_ = 0.07, *p* = 0.94; watch: *t*_(39)_ = 0.02, *p* = 0.98; pen: *t*_(39)_ = 1.22, *p* = 0.23) and in contrast to Experiment 1 there was also no significant difference between the likeability ratings of the three individual items across the groups [ANOVA—*F*_(2,80)_ = 1.73, *p* = 0.18]. As in Experiment 1 there was a high IOS score for mother-owned items in the two groups compared to classmate indicating the presence of an extended self which includes mother.

**Table 2:** **Age and questionnaire scores for 2^nd^ Experiment (mean ± SEM)**.

Measurements	Placebo	Oxytocin	*t*-value	*p*-value
Age (years)	23.00 ± 0.60	22.65 ± 0.41	0.48	0.63
NEO-Five Factor Inventory (NEO-FFI)				
Neuroticism	29.89 ± 1.49	31.85 ± 1.40	0.96	0.35
Extraversion	41.26 ± 1.14	43.20 ± 1.43	1.05	0.30
Openness to experience	40.37 ± 1.05	39.65 ± 1.21	0.45	0.66
Agreeableness	42.74 ± 0.98	42.00 ± 0.83	0.57	0.57
Conscientiousness	43.16 ± 0.73	43.45 ± 1.02	0.23	0.82
Positive and Negative Affective Scale (Positive)	30.74 ± 1.56	29.65 ± 1.37	0.52	0.60
Positive and Negative Affective Scale (Negative)	15.68 ± 1.17	17.00 ± 1.33	0.74	0.46
State-Trait Anxiety Inventory (STAI)—State	36.63 ± 1.90	38.05 ± 1.83	0.54	0.59
State-Trait Anxiety Inventory (STAI)—Trait	43.21 ± 1.43	46.05 ± 1.64	1.30	0.20
Self-Esteem Scale (SES)	32.00 ± 0.84	32.50 ± 1.01	0.38	0.71
Self-Construal Scale (SCS)—Independent	58.37 ± 1.38	57.10 ± 1.04	0.74	0.47
Self-Construal Scale (SCS)—Interdependent	64.00 ± 1.46	61.75 ± 1.67	1.01	0.32
Inclusion of Others in Self (IOS)—Mother	5.63 ± 0.28	5.90 ± 0.22	0.77	0.45
Inclusion of Others in Self (IOS)—Classmate	2.89 ± 0.21	2.65 ± 0.21	0.82	0.42

Similar to the behavioral results of Experiment 1, the indifference point (WTA-WTP) was greater for self- [*t*_(38)_ = 4.44, *p* < 0.001, Cohen’s *d* = 0.84] and mother- [*t*_(38)_ = 5.08, *p* < 0.001, Cohen’s *d* = 0.97], but not for classmate- [*t*_(38)_ = 0.99, *p* = 0.327] or stranger-owned [*t*_(38)_ = 0.09, *p* = 0.927] items in both groups. A two-way ANOVA revealed a significant main effect of ownership [*F*_(3,111)_ = 20.25, *p* < 0.001, partial *η*^2^ = 0.35] and treatment [*F*_(1,37)_ = 4.24, *p* = 0.047, partial *η*^2^ = 0.103] although once again there was no significant interaction [*F*_(1,37)_ = 0.206, *p* = 0.892; see Figure 2B). As in Experiment 1 there was no significant difference between the contribution of the three different items to the overall size of the endowment effect (ANOVA—*F*_(2,76)_ = 0.20, *p* = 0.82). There were no significant correlations between the size of the endowment effect in the two groups and either self-esteem or SCS scores.

To establish whether there were any dose dependent effects of OXT, we carried out a separate analysis using the common conditions in the two experiments with treatment, including dose, (40IU OXT/24IU OXT/PLC—PLC groups from the two experiments were combined) as the between subject factor and ownership (self/mother/classmate) as the within subject one. Results confirmed main effects of ownership [self/mother/classmate, *F*_(2,142)_ = 41.59, *p* < 0.001, partial *η*^2^ = 0.37] and treatment [*F*_(2,71)_ = 4.85, *p* = 0.01, partial *η*^2^ = 0.12] due to the size of endowment effect being increased by both OXT doses compared with PLC (24 IU, *p* = 0.043; 40IU, *p* = 0.034). However, there was no significant difference between 24IU OXT and 40IU OXT (*p* = 1), and no treatment × ownership interaction [*F*_(4,142)_ = 0.32, *p* = 0.86]. Thus, there was no evidence for a dose-dependent effect of OXT.

#### fMRI Results

**Figure 3:**
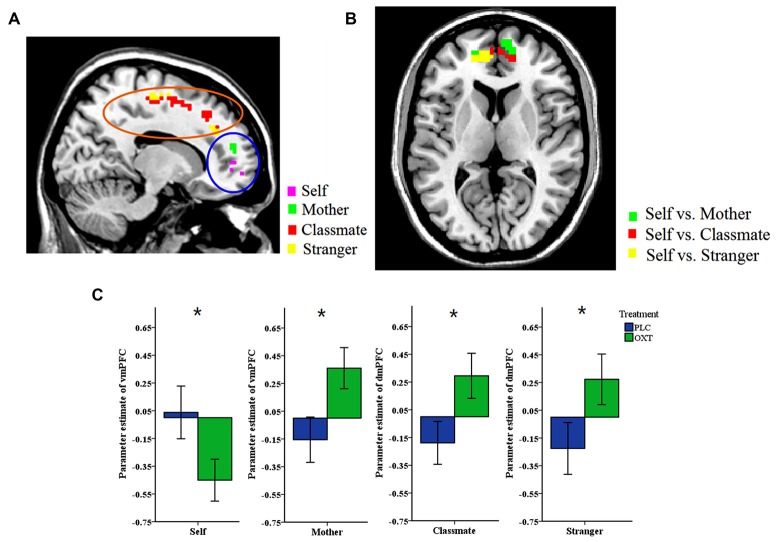
**Whole-brain analysis. (A)** The two brain images show the contrast between the OXT and PLC groups in self-, and mother-ownership conditions (main image) and classmate- and stranger-ownership conditions (smaller image), respectively. Ventral mPFC (vmPFC) activation was decreased by OXT in the self-condition (PLC > OXT) but increased in the mother-condition (OXT > PLC). For dorsal mPFC (dmPFC) OXT increased activation in both classmate and stranger- conditions (OXT > PLC). Display threshold, *p* < 0.05, small volume corrected, voxels >10. **(B)** The contrast between self vs. all others (mother/classmate/stranger) across two groups. OXT reduced the difference in vmPFC activation between self and all others (PLC > OXT). Display threshold, *p* < 0.05, small volume corrected, voxels > 10. **(C)** Histograms show corresponding mean ± SEM changes in parameter estimates in OXT and PLC groups for self and mother in the vmPFC and classmate and stranger in dmPFC (*p < 0.05).

A whole brain analysis was first performed for activation changes in both the price stage (where the price of the object was shown) and the response phase. Significant effects of either ownership or treatment were only found in the price phase. Significant main effects of ownership were found in IFG (−51, 32, −5), middle frontal gyrus (MFG, −33, 23, 37/−48, 17, 43), superior frontal gyrus (SFG, −6, 26, 55), mPFC (−6, 26, 55/−6, 35, 43) and a number of other regions (see Table 3). However, there was no interaction between treatment and ownership (*p* < 0.05, FDR corrected). We found a significant main effect of treatment with (SVC, *p* < 0.05) for a cluster of mPFC voxels (OXT > PLC, see Table 3). However, two sample *t*-tests between the OXT and PLC groups showed that vmPFC activation was decreased by OXT in the self- condition (PLC > OXT) but increased in the mother- condition (OXT > PLC). On the other hand for dmPFC, OXT increased activation in both classmate and stranger conditions (OXT > PLC) with SVC for mPFC voxel-corrected (see Table 3 and Figure 3). The contrast self vs. all others (mother/classmate/stranger) revealed that OXT reduced the difference in vmPFC activation between self and all others (PLC > OXT) with SVC for mPFC voxel corrected (see Table 3). In addition, the contrast self vs. remote others (classmate and stranger) revealed that OXT reduced the difference between self and others (i.e., excluding mother) in both vmPFC and dmPFC (PLC > OXT) with SVC for mPFC voxel corrected (see Table 3).

**Table 3:** **Areas of brain activation during price stage (MNI coordinates)**.

Brain Region	BA	No. Voxels	Peak *t*-value	*x*	*y*	*z*
**Main effect of ownership**						
L. Inferior Frontal Gyrus	45	99	13.75	−51	32	−5
L. Superior Frontal Gyrus	8	64	8.07	−6	26	55
Medial Prefrontal Cortex			7.17	−6	35	43
Medial Prefrontal Cortex			6.86	−3	41	37
L. Dorsal Medial Prefrontal Cortex	9	15	7.54	−33	23	37
L. Dorsal Medial Prefrontal Cortex	9	11	6.50	−48	17	43
**Main effect of treatment (OXT > PLC)**						
R. Dorsal Medial Prefrontal Cortex	9	17	2.22	12	38	31
**Self ownership (PLC > OXT)**						
R. Ventral Medial Prefrontal Cortex	10	10	2.52	9	56	−11
R. Ventral Medial Prefrontal Cortex	10	15	2.21	15	50	4
L. Ventral Medial Prefrontal Cortex	10	24	2.38	−6	50	−8
L. Ventral Medial Prefrontal Cortex	10	39	2.50	−18	56	7
**Mother ownership (OXT > PLC)**						
R. Ventral Medial Prefrontal Cortex	10	10	2.54	15	50	19
L. Ventral Medial Prefrontal Cortex	10	20	3.40	−21	47	16
**Classmate ownership (OXT > PLC)**						
R. Dorsal Medial Prefrontal Cortex	9	16	2.07	12	38	31
R. Medial Prefrontal Cortex	8	10	2.16	15	29	43
**Stranger ownership (OXT > PLC)**						
R. Dorsal Medial Prefrontal Cortex	9	17	2.22	12	38	31
**Self vs. Mother (PLC > OXT)**						
R. Ventral Medial Prefrontal Cortex	10	27	2.13	12	59	4
L. Ventral Medial Prefrontal Cortex	10	63	2.92	−15	47	7
**Self vs. Classmate (PLC > OXT)**						
L. Ventral Medial Prefrontal Cortex	10	49	2.47	−6	50	10
R. Ventral Medial Prefrontal Cortex	10	29	2.07	12	50	7
**Self vs. Stranger (PLC > OXT)**						
L. Ventral Medial Prefrontal Cortex	10	20	2.40	−24	44	10
**Self vs. Remote others (Classmate & Stranger, PLC > OXT)**						
L. Ventral Medial Prefrontal Cortex	10	47	2.37	−24	44	10
				−6	50	7
L. Dorsal Medial Prefrontal Cortex	9	10	2.30	−15	14	52
L. Dorsal Medial Prefrontal Cortex				−12	35	46
		20	2.14	−9	44	43
R. Dorsal Medial Prefrontal Cortex	9	10	1.85	9	50	19

*Brain regions exhibiting significant activation changes during the endowment effect task following a whole-brain analysis (*p* < 0.05 FDR corrected). The table gives anatomical regions, significance of activation changes, their laterality [left (L), right (R), or bilateral], and BA, Brodmann’s areas*.

**Figure 4:**
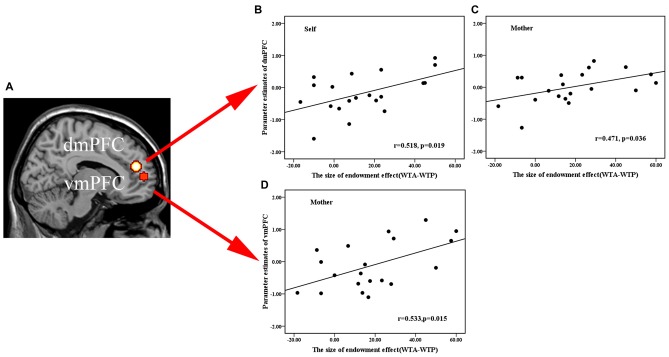
**Regions of interest (ROIs) analysis. (A)** Brain activation was extracted from 8 mm radius spherical dmPFC (−6, 46, 20) and vmPFC (−18, 56, 7) ROIs. **(B,C)** Scatterplots of a regression analysis between neural parameter estimates of dmPFC and behavioral indifference point (disparity between WTA and WTP) in self- **(B)**, mother-conditions **(C)** and between the behavioral performance in mother-condition and neural activations of vmPFC **(D)**. The averaged beta-weights for each subject were extracted from the voxels in the same ROIs where such correlation was significant in the random effects group analysis.

**Figure 5:**
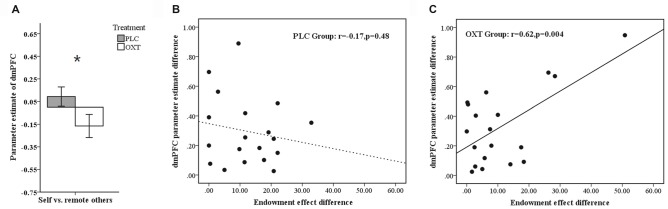
**(A)** Histogram shows mean ± SEM parameter estimates for the contrast self vs. remote others in the dmPFC in OXT and PLC groups (*p < 0.05). **(B,C)** Regression plots showing correlations between the activation difference in the dmPFC for the self vs. remote others condition and the difference in the size of the endowment effect between self and remote others for subjects in the PLC **(B)** and OXT **(C)** groups. In both cases absolute values are plotted.

To test whether there was relationship between activation in the mPFC ROIs and the size of a subject’s endowment effect, we performed a Pearson correlation analysis in all ownership conditions. Results showed that activation in the dmPFC (−6, 46, 20) ROI in the OXT, but not the PLC, group was significantly positively correlated with the size of endowment effect for self and mother ownerships but not for classmate or stranger (OXT: self—*r* = 0.518, *p* = 0.019; mother—*r* = 0.471, *p* = 0.036; classmate—*r* = 0.315, *p* = 0.176; stranger—*r* = 0.372, *p* = 0.106; PLC: self—*r* = 0.276, *p* = 0.252; mother—*r* = −0.082, *p* = 0.74; classmate—*r* = 0.178, *p* = 0.465; stranger—*r* = 0.108, *p* = 0.66, see Figures 4A–C), although Fisher’s *z*-transformation did not reveal a significant difference between the two treatment groups (self: *z* = 0.83, *p* = 0.40; mother: *z* = 1.70, *p* = 0.088; classmate: *z* = 0.42, *p* = 0.67; stranger: *z* = 0.81, *p* = 0.42). On the other hand, the vmPFC (−18, 56, 7) ROI was significantly positively correlated with the size of endowment effect in mother- but not self-, classmate- or stranger-ownership conditions only in the OXT group (OXT: self—*r* = 0.195, *p* = 0.410; mother—*r* = 0.533, *p* = 0.015; classmate—*r* = 0.077, *p* = 0.746; stranger—*r* = 0.292, *p* = 0.212; PLC: self—*r* = 0.139, *p* = 0.571; mother—*r* = 0.146, *p* = 0.55; classmate—*r* = 0.151, *p* = 0.538; stranger—*r* = −0.112, *p* = 0.647, see Figures 4A,D), although again Fisher’s *z-transformation* did not reveal a significant difference between the two groups (*z* = 1.28, *p* = 0.20). Moreover, the dmPFC (−15, 14, 52) ROI based on absolute values from the contrast between self vs. remote others (classmate and stranger) was significantly positively correlated with the corresponding difference in the size of the endowment effect only in OXT group (OXT: *r* = 0.622, *p* = 0.004; PLC: *r* = −0.171, *p* = 0.484) with Fisher’s *z-transformation* showing that the difference between the two groups was significant (*z* = 2.55, *p* = 0.01; see Figure 5). Thus in the OXT group the smaller the activation difference in dmPFC between items owned by self and remote others, the smaller was the difference in the size of the endowment effect between them.

**Figure 6:**
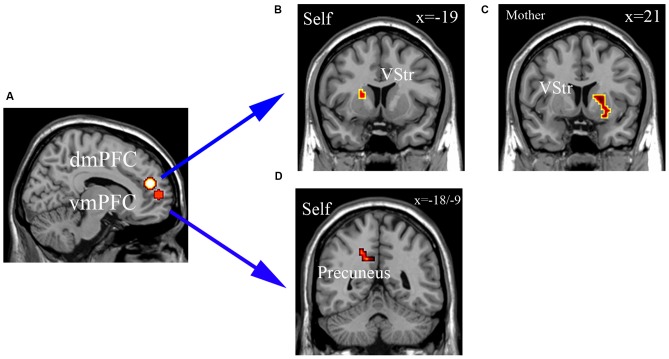
**The generalized psychophysiological interaction (gPPI) analysis. (A)** dmPFC (−6, 46, 20) and vmPFC (−18, 56, 7) as seed regions. **(B,C)** dmPFC (−6, 46, 20) functional connectivity with the left ventral striatum (VStr; −19, 11, 10) was decreased in the self-ownership condition for OXT compared with PLC **(A)**, and for dmPFC and right VStr (21, 11, −5) in the mother-ownership condition **(B)**. **(D)** OXT decreased the functional connectivity between vmPFC (−18, 56, 7) and precuneus (−18, −55, 37/−9, −49, 31) in the self-ownership condition. Display threshold, *p* < 0.05, small volume corrected, voxels >10.

The gPPI analysis revealed that the dmPFC (−6, 46, 20) functional connectivity with the left ventral striatum (VStr; −19, 11, 10) was decreased in the self-ownership condition for OXT compared with PLC groups and for dmPFC and right VStr (21, 11, −5) in the mother-ownership condition (see Figures 6A–C). There were no effects for the classmate- or stranger-ownership conditions. OXT also decreased the functional connectivity between vmPFC (−18, 56, 7) and precuneus (−18, −55, 37/−9, −49, 31) in the self-ownership condition, but not in mother-, classmate-, or stranger-ownership ones (see Figures 6A,D). However, neither the strength of dmPFC-VStr (−0.192 < *r* < 0.352, *p* > 0.128) nor vmPFC-precuneus (−18, −55, 37; −0.287 < *r* < 0.162, *p* > 0.234 and −9, −49, 31; −0.319 < *r* < −0.083, *p* > 0.182) functional connectivities were correlated with the size of endowment effect in all ownership conditions across the two groups. There were no significant correlations in either the OXT or PLC groups between self-esteem or SCS scores and functional connectivity changes.

## Discussion

The present study investigated whether the effects of OXT on reducing the distinction between self and other which has previously been observed in the context of personal attributes (Zhao et al., 2016), pain empathy (Abu-Akel et al., 2015) and preference for communal situations (Bartz et al., 2015) extends to objects possessed by self and others using an endowment task. The behavioral results from two independent experiments showed a main effect of OXT whereby it significantly enhanced the strength of the endowment effect in individuals from a collectivist culture for both self- and all categories of other- (close: mother/father and remote: classmate/stranger) owned items. Neuroimaging results showed a differential pattern of OXT-evoked changes in the dmPFC and vmPFC with it decreasing activation in the vmPFC for self-owned items but increasing it for mother-owned ones in vmPFC and increasing it in the dmPFC for classmate and stranger-owned items. Activation changes in the dmPFC for self- and mother-owned items were associated with the magnitude of the endowment effect as were differences in activation between self- and remote other-owned items. Additionally, functional connectivity between the dmPFC and the VStr was reduced by OXT in both self and mother-conditions suggesting that self- and mother-owned items may have influenced reward processing. There was also reduced functional connectivity between dmPFC and the precuneus in the self-ownership condition which might further indicate a reduced importance of self-owned items.

The endowment effect can no longer solely be attributed to a traditional loss aversion account (Morewedge et al., 2009; Morewedge and Giblin, 2015), and in the current study it is considered more to reflect the tendency to value self over others (Beggan, 1992; Gawronski et al., 2007; Dommer and Swaminathan, 2013) and increasing the positivity or decreasing the negativity of the self is a means of maintaining this (Brown and Dutton, 1995). Belk (1988) has argued that possessions are an important component of sense of self with an association between a possession and self being created by virtue of ownership. On the other hand evidence suggests that “shared representations” between self and other lie at the core of the phenomena of affective sharing (Bartz et al., 2010). Our current results confirm those of our previous study (Feng et al., 2013) that in subjects from a Collectivist culture shared representations in the context of the endowment effect extend to close relatives, such as mother and father. The behavioral effect of OXT treatment in both experiments carried out was to generally enhance the value placed on both self and all other-owned items, which suggests that it is acting both to further promote the shared representation between self and close others and additionally between self and remote others. On the other hand, the bias towards placing a greater value on self- and mother-owned items compared to remote-other owned ones is still maintained under OXT. This is in general agreement with our previous findings of a main effect of OXT on decreasing response times for making trait-judgments for self as opposed to close or remote others, although in contrast to the current study OXT also abolished the normal self-bias in the accuracy of making trait-judgments (Zhao et al., 2016). Thus, together with findings showing that OXT promotes a more other-orientated social responses (Abu-Akel et al., 2015; Bartz et al., 2015), our current and previous results suggest that it generally facilitates a more extensive shared representation of self both in terms of the social attributes and feelings of others as well as their possessions. Such a shared representation of self might also lead to greater affiliation with others and thereby contribute to the well-established effects of OXT in animal models and humans in promoting the formation and maintenance of social bonds (see Striepens et al., 2011; Hurlemann and Scheele, 2016).

The mPFC is involved in tasks requiring self-evaluation and reflection (Kelley et al., 2002; Moran et al., 2006; Feng et al., 2013). Our current fMRI findings both confirm and extend our previous results showing that the mPFC is the key region where OXT alters activity in association with its behavioral effects in influencing both self and other processing (Zhao et al., 2016). Indeed, the whole brain analysis only revealed both main effects of OXT and ownership in medial prefrontal regions and there were no effects in other brain regions at *p* < 0.05 FDR corrected. Overall, in the current study OXT increased mPFC activation across conditions whereas for the trait-judgment task it produced an overall decrease (Zhao et al., 2016). In line with our previous finding using a trait-judgment paradigm OXT altered activity in both dmPFC and vmPFC, although while similar reductions in activation were seen for self-owned items in vmPFC a notable difference in the current study was that vmPFC activation was increased for mother-owned items and dmPFC activation was increased for classmate and stranger-owned ones. Thus in the context of the endowment effect OXT increased mPFC activity in all three other-ownership conditions whereas for trait-judgments it decreased it. The dmPFC, activation for both self- and mother-owned items was positively associated with the size of the endowment effect in the OXT group. However, the absolute activation difference between self- and remote other-owned items was also positively associated with the size of the difference in the endowment effect between them (i.e., the smaller the dmPFC activation difference between self and remote others the smaller the difference in the size of their respective endowment effects). Thus OXT’s facilitation of the endowment effect in both self and others appears to be associated with a reduced difference between dmPFC activation in self- and other-owned conditions. In our previous study we also found that mPFC activation during the endowment task was positively correlated with selling price (Feng et al., 2013). On the other hand, for the vmPFC there is a positive association with the size of the extended endowment effect observed for mother-owned items, with OXT effectively promoting a larger difference in activation between self and close others. The vmPFC is activated during tasks involving self-knowledge and person perception (Amodio and Frith, 2006) and has recently been proposed to be particularly associated with the personal importance assigned to self-representations and with aspects of self which have high personal value such as possessions and close others (D’Argembeau, 2013). Thus OXT may have additionally influenced the size of the endowment effect for the possessions of close others (i.e., mother-owned items) by acting on the vmPFC.

Overall therefore the main effect of OXT on increasing the size of the endowment effect for both self and close and remote others may have been contributed to by its differential actions on the dmPFC and vmPFC. Thus OXT may act primarily via the vmPFC to increase the size of the endowment effect in close others (mother) whereas for remote others (classmate and stranger) its effect on the dmPFC appears to be more involved. The relative contributions of the dmPFC and vmPFC to the increase in the size of the endowment effect in the self-condition under OXT appears to be more complex however. While the decreased vmPFC response might indicate a reduction in the size of the endowment effect in the self-condition, there is at the same time a reduced difference between the vmPFC response in the self-condition and that in all three of the other-conditions compared to the PLC group. Thus OXT may overall have acted to increase the size of the endowment effect in the self-condition via the vmPFC despite its overall effect being to reduce vmPFC activity. On the other hand, in the dmPFC the magnitude of the response in the self-condition is positively associated with the size of the endowment effect and at the same time OXT is again acting to decrease the difference between self and remote-others. Thus OXT may also be acting at the level of the dmPFC to increase the size of the endowment effect both in the self- and remote other-conditions.

The functional connectivity analysis showed that OXT treatment reduced the strength of the connectivity between dmPFC and VStr for both self- and mother-owned items, although in neither case was there a significant association with the size of the endowment effect in the two conditions, and the corresponding dmPFC BOLD response was also not significantly altered. The VStr processes information on expected reward magnitude to guide adaptive action preparation and reward learning, and the computation of anticipated gains or expected values (Knutson et al., 2007; Brown and Cai, 2010). Possibly a reduced functional connectivity from the dmPFC to the VStr might therefore reflect an action of OXT in decreasing the reward or valuation of self- and close-other items, thereby enhancing that for remote other-owned items. However, since behaviorally OXT acted to increase the size of the endowment effect in both self- and mother-owned conditions, suggestive of enhanced reward or value, the reduced functional connectivity observed could instead reflect an increase in their reward or value as a result of reduced negative feedback from the VStr to the dmPFC, since we cannot identify the specific direction of the observed functional connectivity change between these two regions. On the other hand the reduced functional connectivity between the vmPFC and the precuneus in the self-owned condition occurred in association with a corresponding reduced vmPFC BOLD response. The functional connectivity change in this case may therefore primarily be driven by the reduction in the BOLD response with both activation and functional connectivity decreases combining to produce a reduced self-bias, the precuneus along with the vmPFC being known to play a role in self-processing (Cavanna and Trimble, 2006).

In contrast to our previous study investigating the effects of OXT on trait-judgments, we found no correlation between its behavioral or neural effects and levels of self-esteem in the endowment task. Another previous study has also reported that activation in the anterior cingulate in response to processing of self-referential items was negatively associated with trait levels of self-esteem (Yang et al., 2012). Since the magnitude of self-esteem tends to have a greater influence in contexts where social rejection is a potential factor (Yang et al., 2012), it is possible that this is of less relevance when judging the value of possessions owned by self or others than in the more personal context of attributing positive or negative behavioral traits to self and others. We also found no evidence for a modulatory influence of SCS scores on behavioral or neural effects of OXT and so it would appear that the relative strength of collectivism compared to independent traits within our cohort of Chinese subjects was not of great importance. It would clearly be of interest however to confirm in future studies whether similar effects of OXT on the endowment effect occur in independent cultures in order to establish whether there are modulatory effects of an independent vs. collectivist orientation.

The present study has several limitations. First, we found no evidence for dose-dependent effects of OXT in the endowment task. Since we chose to use two OXT doses which have both previously been reported to have significant functional effects (Striepens et al., 2011; Guastella et al., 2013) it is possible that this impaired our chances of demonstrating clear dose-dependent effects which might require inclusion of lower doses with weaker functional effects. Additionally, small timing and other differences in task presentation and scanner noise in the second experiment may have also contributed. Second, we only included male subjects in the study to avoid potential issues for controlling for menstrual cycle effects. However, there is growing evidence for sex-dependent behavioral and neural effects of OXT (see Gao et al., 2016) and thus we cannot exclude the possibility that there may also be sex-dependent effects of OXT in relation to self-processing and the endowment effect. Thirdly, in the current study the anxiety/mood assessments were only made prior to treatment and so we cannot exclude the possibility that a non-specific OXT effect on mood/anxiety might have influenced subjects’ behavior. However, a number of our previous studies and those by other groups have reported no measureable influence of intransal OXT treatment on either state anxiety or positive or negative mood (see Scheele et al., 2015; Tabak et al., 2016; Xu et al., 2017).

In summary, the current study has shown in two separate experiments that OXT enhances the value placed on possessions owned by both self and others in the context of an endowment effect task. Thus OXT effects on self- and-other processing are not limited to personal attributes. The behavioral effects of OXT on the value placed on possessions also appear to involve differential actions on both the dmPFC and vmPFC and modulating their functional connections with reward and other self-processing regions. Taken together these findings provide further support for a role of OXT in reducing a self-other distinction.

## Author Contributions

WZ and KMK designed the experiments; WZ, YG and LL conducted the experiments; WZ, ZZ, XM, LX and SY analyzed the data; WZ, YG, LL, ZZ, XM and LX drafted the manuscript, WZ and KMK revised the article. All authors reviewed the manuscript and agree to be accountable for the content of the work.

## Funding

This work was supported by National Natural Science Foundation of China (NSFC) grant number: 31530032.

## Conflict of Interest Statement

The authors declare that the research was conducted in the absence of any commercial or financial relationships that could be construed as a potential conflict of interest.

## Footnotes

^1^http://www.fil.ion.ucl.ac.uk/spm/software/spm8/
